# Are older women likely to use medicines than older men? (Results from AHAP study)

**Published:** 2014

**Authors:** Ali Bijani, Ali Reza Hasanjani Roshan, Seddiqah Yazdanpour, Seyed Reza Hosseini

**Affiliations:** 1 Social Determinants of Health Research Center, Babol University of Medical Sciences, Babol, Iran.; 2 Infectious Diseases Research Center, Babol University if Medical Sciences, Babol, Iran.; 3 Babol University of Medical Sciences, Babol, Iran.

**Keywords:** Drug, Elderly, Sex, Self medication, Disease

## Abstract

***Background: ***The health of elderly population in the world has been an important issue in recent century and the use of appropriate or inappropriate medications is challenging among them. The purpose of this study was to assess the pattern of medication in elderly population in Amirkola, northern of Iran.

***Methods:*** This study was conducted on 1534 elderly subjects who participated in Amirkola Health and Ageing Project (AHAP) in 2013. The number of drugs that was used regardless of their indication in terms of age, level of education, disease, cognitive or depression and social support were recorded and compared in both sexes.

***Results:*** The mean number of drugs used in men and women was 2.1±2.45 and 3.59±2.75, respectively (P=0.000). Concurrent use of > 4 drugs was seen in 16.5% of men and in 35.12% of women (P=0.000). The difference for using the number of drugs was significant between sexes with low educational level, but was similar in educated individuals. The use of polypharmacy was associated with the number of concurrent diseases (r=0.58, P=000), cognitive status (r=0.065, P=0.012), social support (r=-0.1, P=0.008), and depression (r=0.273, P=0.000).

***Conclusion: ***The results show that the use of polypharmacy in the elderly population in Amirkola is relatively high and they need to be educated. Considering the other indices, this problem highly manifested more in the elderly women.

Elderly individuals are considered and constitute one of the major public health challenges we encounter today (1). The percentage of this population over 60 years in Iran is increasing and somehow the increasing scale in 1986 was 5% reaching over 7% in 2005 and has now increased more. In the USA, an individual > 65 years in 1900 was 4.1% and reached to 12.6% in 2000 (2). In 2006, the Iranian population over 60 years was reported to be 7.4% (3). The population over 60 years is estimated to be 10% in the next 25 years (4). Studies revealed that elderly persons are at increasing risk of the development of organic and psychological problems that need several drugs simultaneously for their treatment (1, 5-7). An elderly subject with age over 70 years may have more than three chronic diseases that certainly increase the number of drugs to be used for his/her treatment (2). On the other hand, habit forming use of drugs, self medication, administration of inappropriate drugs, polypharmacy and co-pharmacy are challenging problems that attracted researchers to this field (6, 7). The World Health Organization (WHO) recommended polypharmacy for those who take 5 or overmedications per day (8).

A population-based survey in 2005-2006 in elderly in the USA showed that 37.1% of men and 36% of women took at least five different drugs (9). Since a cohort study has been started in Amirkola, we tried to assess the pattern and number of drugs used between males and females in the community of Amirkola. 

## Methods

In this study, based on the data gathered in elderly individuals in Amirkola, in 1616 subjects, the number of drugs that they regularly received was recorded during one month in 2013 (10). In this survey, we did not judge the kind of drug, dose, indication for using the drug, and interaction between drugs, but we considered the number of drugs they used as a variable. The demographic data like age, sex, level of educations were recorded. 

Twenty five diseases such as cardiovascular, pulmonary, digestive, liver, renal and CVA disorders were also considered in this study. Cognitive status, presence of depression and social support were assessed using standard questionnaire of MMSE, GDS and DSSI. Statistical analysis was done using SPSS version 20. The relation with all the noted variables with the number of drugs was assessed. T-test, chi-square test, analysis of variance, Pearson coefficient test and linear regression model were used. P<0.05 was considered significant.

## Results

**Figure 1:**
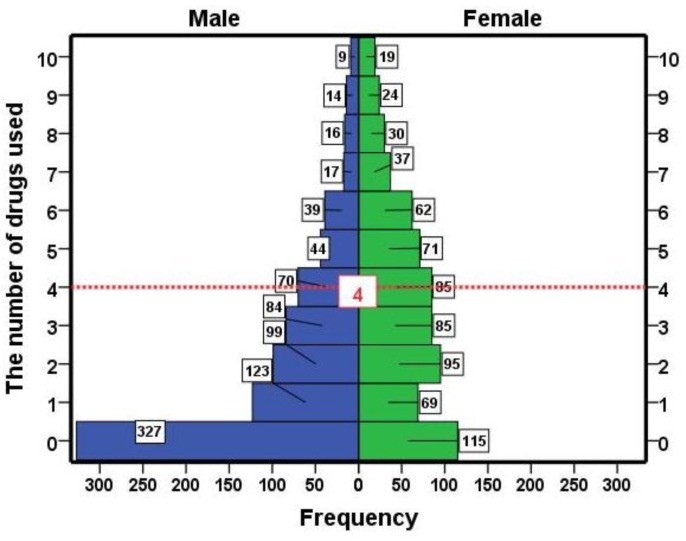
Frequencies of the number of in elderly individuals in Amirkola

**Figure 2:**
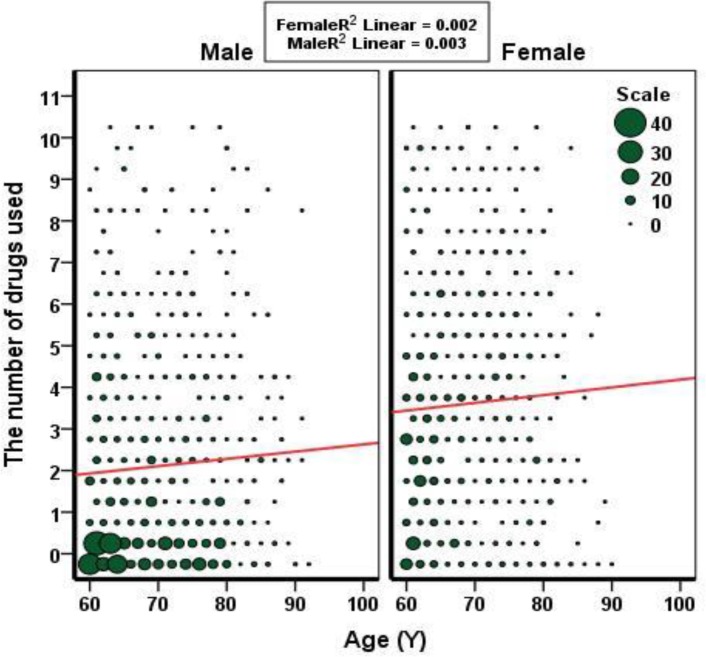
The relation between ages with the number of drugs used regarding sexes

**Figure 3:**
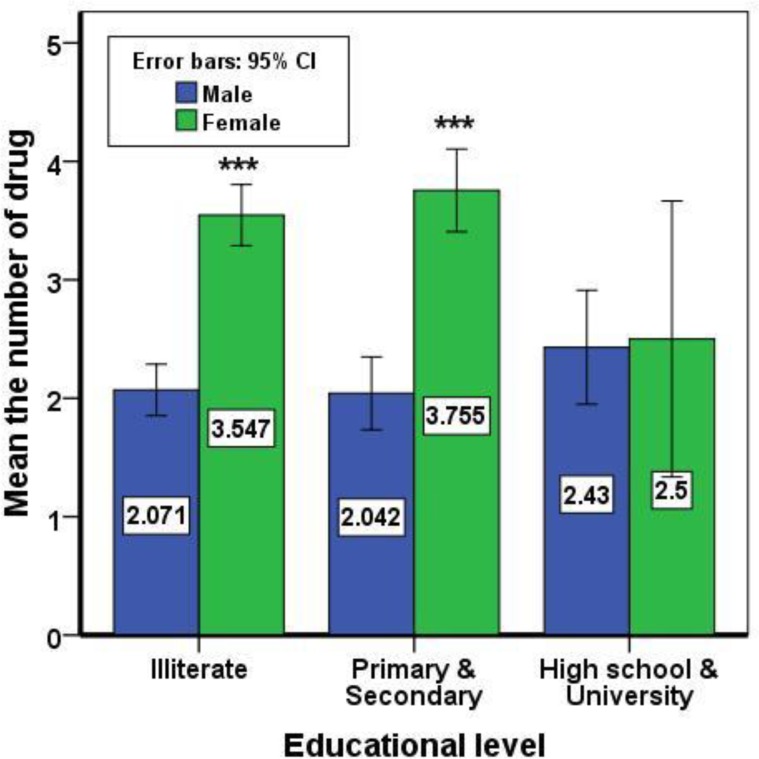
The relation between educational levels with the number of drugs used regarding sexes

The data from drug use in 82 elderly individuals were incomplete and excluded from the study. So, the data from 1534 cases were analyzed. Among them, 842 (54.9%) were males and 692 (45.1%) females. The mean age of these subjects was 69.22±7.35 years. The mean number of drug used in men and women was 2.1±2.45 and 3.59±2.75, respectively (P=0.000). Figure 1 shows the distribution of drugs used in both sexes. With increasing age, the number of used drugs slightly increased but not significant (r=0.023, p=0.374). These findings were similar in both sexes (figure 2). Simultaneous use of > 4 drugs in men 16.51% (95% CI, 14-19.02) and in women 35.12% (95% CI, 31.55-38.68) (P=000). There was significant difference in several drugs used regarding educational level between two sexes, but those with high school educational level was similar (figure 3). Under the supervision of the physician, 66.8% of the cases received medications and only 21% of subjects got special drugs from the pharmacy.

The mean simultaneous disease among 25 subjects was 2.7±1.96 and 11.3% of elderly individuals were ill and one case had 12 different diseases. Concurrently, receiving of more drugs were significant regarding simultaneous multiple diseases (r=0.58, P=0.000), cognitive status (r=0.065, P=0.012), social support (r=-0.1, P=0.008) and depression (r=0.273, P=0.000). Table 1 shows linear regression model predicting factors of the number of drugs used.

**Table 1:** Linear regression model predicting factors of the number of drugs used

**Model**	**Unstand** **ardized Coefficients ** **B**	**95.0% Confidence Interval for B**		**Standardized Coefficients** **Beta**	**T**	**P.value**
		**Lower Bound**	**Upper Bound**			
(Constant)	-1.732	-3.598	.135		-1.820	.069
Age (year)	.018	.002	.033	.048	2.209	.027
Sex (Female)	.512	.266	.758	.094	4.082	.000
MMSE	-.004	-.017	.009	-.013	-.615	.539
GDS	.038	.001	.075	.048	2.004	.045
DSSI	.018	-.020	.055	.021	.923	.356
Diseases (N)	.738	.676	.799	.535	23.497	.000

## Discussion

In this study, we found that the mean number of drugs used in men was 2.1±2.45 and in women was 3.59±2.75, thus women used more drugs than men used in this society. Polypharmacy was seen in 16.51% men and in 35.12% of women (P=0001). In 2009, 63% of Canadian elderly individuals were taking more than 5 medications and 30% of those older than 85 years were taking more than 10 that are higher than that the results of our study (11). In Sweden, those taking 5 or more medications had a 6.2% increase in prescription drug expenditure and those taking 10 or more medications had a 7.3% increase (12). In Goiania, Brazil, women used more medicines than men (3.94 and 3.06, respectively) and the prevalence of practicing polypharmacy was 26.4% that is similar to the findings of our study (13). Greater use of medication by elderly women may be related to longer living of women than men that predispose them with chronic illness, and more attention to their health and also more visitations in health centers. In contrast, the mean drug used in charity foundation for elderly individuals were between 5-10 drugs in the developed countries and the resident of charity in the developing countries were reported to be between 41% to 58.6% (14-20). These differences might be due to more chronic diseases of this population cared in the charity.

In this study, there was significant difference in several drug use regarding educational levels between two sexes, and those with high educational degrees used less drugs. Similar results were obtained by other researchers (13). In the present study, 66.8% patients received their drugs under the supervision of the physician, and only 21% of subjects got special drugs from the pharmacy without a doctor’s prescription. Even in developed countries, unnecessary drugs were studied in 128 older male outpatients and showed that 58.6% of patients took one or more unnecessary prescribed drugs (21). It is also shown that elderly population use one unprescriped drug after consuming each 2-3 prescribed drugs (22). Another study showed that drugs without prescription are used in 97% of regimen of therapy in elderly (23). Self-medication places the health of the elderly population at risk. This practice can increase risks related to prescribed medicines, delay correct diagnosis and mask illness even with adverse effects (13).

Polypharmacy is often necessary, as many elderly people suffer from a variety of illnesses and symptoms which require the use of various medicines to guarantee the best possible quality of life. In this study, we found that using polypharmacy (high figures of drugs) was associated with the number of concurrent diseases, cognitive status, social support, and depression. Similar results were obtained by other researchers (8, 9, 24). In a prospective study of 294 elderly subjects, 22% of patients taking 5 or less medications had impaired cognition (3). The weakness of this study may be because of the lack of list of the drugs used by our cohort elderly individuals.

 It is necessary for health care professionals to find out the strategies in this elderly individuals supportive of their own efforts to reduce polypharmacy. In summary, the results of our study show that the use of drugs in the elderly population in Amirkola is relatively high and they need to be educated. Considering the other indices, this problem was higher in the elderly women.

## Notes

**Funding: **The Deputy Research of Babol University of Medical Sciences supported the funding (Grant Number 9236318).

## Notes

**Conflict interest: **All authors state no conflict of interests.
